# Environmental Burden of Disease in Europe: Assessing Nine Risk Factors in Six Countries

**DOI:** 10.1289/ehp.1206154

**Published:** 2014-02-28

**Authors:** Otto Hänninen, Anne B. Knol, Matti Jantunen, Tek-Ang Lim, André Conrad, Marianne Rappolder, Paolo Carrer, Anna-Clara Fanetti, Rokho Kim, Jurgen Buekers, Rudi Torfs, Ivano Iavarone, Thomas Classen, Claudia Hornberg, Odile C.L. Mekel, the EBoDE Working Group.

**Affiliations:** 1 National Institute for Health and Welfare (THL), Department of Environmental Health, Helsinki, Finland; 2 National Institute of Public Health and the Environment (RIVM), Bilthoven, Netherland; 3 French Institute for Public Health Surveillance (InVS), Saint-Maurice, France; 4 Federal Environment Agency (Umweltbundesamt, UBA), Berlin/Dessau-Roßlau, Germany; 5 Department of Occupational and Environmental Health, University of Milan, Milan, Italy; 6 World Health Organization, Regional Office for Europe, Bonn, Germany; 7 Flemish Institute for Technological Research (VITO), Mol, Belgium; 8 Italian National Health Institute (ISS), Rome, Italy; 9 University of Bielefeld, School of Public Health, Department ‘Environment and Health,’ Bielefeld, Germany; 10 NRW Centre for Health (LZG.NRW), Bielefeld, Germany

## Abstract

Background: Environmental health effects vary considerably with regard to their severity, type of disease, and duration. Integrated measures of population health, such as environmental burden of disease (EBD), are useful for setting priorities in environmental health policies and research. This review is a summary of the full Environmental Burden of Disease in European countries (EBoDE) project report.

Objectives: The EBoDE project was set up to provide assessments for nine environmental risk factors relevant in selected European countries (Belgium, Finland, France, Germany, Italy, and the Netherlands).

Methods: Disability-adjusted life years (DALYs) were estimated for benzene, dioxins, secondhand smoke, formaldehyde, lead, traffic noise, ozone, particulate matter (PM_2.5_), and radon, using primarily World Health Organization data on burden of disease, (inter)national exposure data, and epidemiological or toxicological risk estimates. Results are presented here without discounting or age-weighting.

Results: About 3–7% of the annual burden of disease in the participating countries is associated with the included environmental risk factors. Airborne particulate matter (diameter ≤ 2.5 μm; PM_2.5_) is the leading risk factor associated with 6,000–10,000 DALYs/year and 1 million people. Secondhand smoke, traffic noise (including road, rail, and air traffic noise), and radon had overlapping estimate ranges (600–1,200 DALYs/million people). Some of the EBD estimates, especially for dioxins and formaldehyde, contain substantial uncertainties that could be only partly quantified. However, overall ranking of the estimates seems relatively robust.

Conclusions: With current methods and data, environmental burden of disease estimates support meaningful policy evaluation and resource allocation, including identification of susceptible groups and targets for efficient exposure reduction. International exposure monitoring standards would enhance data quality and improve comparability.

Citation: Hänninen O, Knol AB, Jantunen M, Lim TA, Conrad A, Rappolder M, Carrer P, Fanetti AC, Kim R, Buekers J, Torfs R, Iavarone I, Classen T, Hornberg C, Mekel OC, EBoDE Working Group. 2014. Environmental burden of disease in Europe: assessing nine risk factors in six countries. Environ Health Perspect 122:439–446; http://dx.doi.org/10.1289/ehp.1206154

## Background

Scientific evidence shows clearly that environmental risk factors affect human health. Properly targeted and followed-up environmental health policies, such as the coal burning ban in Dublin, Ireland, in 1990 (Clancy et al. 2002) and the smoking ban in public places in Rome, Italy, in 2005 (Cesaroni et al. 2008) have demonstrated significant population health benefits.

To develop effective policy measures and focus research efforts, it is important to prioritize environmental risk factors based on their health impact. Environmental burden of disease (EBD) measures can be used to express diverging health effects in one unit, such as disability-adjusted life years (DALYs). DALYs give an indication of the equivalent number of healthy life-years lost in a population due to premature mortality and morbidity (Murray and Lopez 1996).

The Dutch National Institute for Public Health and the Environment (RIVM) conducted one of the first systematic European studies using DALYs to compare the health impact of various environmental risk factors (de Hollander et al. 1999). The study highlighted that only a few top-ranking risk factors produced > 90% of the EBD.

The World Health Organization (WHO) included a ranking of selected environmental exposures in the *World Health Report 2002* (WHO 2003), addressing more than a dozen risk factors from a global point of view (Prüss-Üstün et al. 2003) and providing methodological guidance (WHO 2013). The Organisation for Economic Co-operation and Development (OECD) compared EBD with monetary impacts in the *OECD Environmental Outlook* (OECD 2001). More specific EBD studies have looked at indoor air (De Oliveira Fernandes et al. 2009; Logue et al. 2012), chemicals (Prüss-Üstün et al. 2011), secondhand smoke (SHS) (Öberg et al. 2011), and foodborne pathogens (Havelaar et al. 2012). Some of these studies used expert elicitation (De Oliveira Fernandes et al. 2009; Prüss-Üstün and Corvalan 2006), and others reviewed results from previous studies (Prüss-Üstün et al. 2011) or used a “bottom up” data-driven approach to calculate DALYs (Havelaar et al. 2012; Logue et al. 2012).

In this review, we aimed to test the availability of data and applicability of methods for a data-driven European multinational comparison of the EBD. By looking at the environmental causes of the burden of disease, we provide important information for prioritizing and motivating preventive policies, such as reducing air pollution, traffic noise, and SHS.

## Objectives

The Environmental Burden of Disease in European countries (EBoDE) project aimed to provide harmonized EBD assessments for the countries participating. Specifically, it aimed to

Prioritize selected environmental exposures relevant for the European situation based on their annual health impacts
Make data-driven EBD assessments comparable among countries and among environmental risk factors
Assess variation and uncertainty in the input parameters and results
Assess data availability and method applicability for this type of EBD assessment.

In this review we present an overview of the results of the EBoDE project. We focus on the overall results—comparison of the risk factors. More details about the methodology and data are available in the full EBoDE project report (Hänninen and Knol 2011).

## Methods

The EBoDE project was launched in 2009 at a WHO meeting (WHO 2009a). Below, methods, data, and results are briefly described.

*Selection of environmental risk factors, health end points, and exposure–response functions*. Environmental risk factors were selected by the project group based on known public health impacts, high individual risks, public concern, economic interests, and pragmatic reasons related to data availability. The nine selected risk factors were benzene, dioxins [including furans and dioxin-like polychlorinated biphenyls (PCBs)], SHS, formaldehyde, lead, traffic noise (including road, rail, and air traffic noise), ozone, airborne particulate matter, and radon.

Health end points defined in the *International Classification of Diseases, 10th Revision* (ICD-10; http://www.who.int/classifications/icd/en/) for each risk factor (Table 1) were selected based on WHO systematic reviews, guidelines, and other methods identified in a nonsystematic literature review conducted in 2009 (WHO 2009a) as part of the current work (see references in Table 1). Exposure–response functions were selected from international recent meta-analyses, WHO guidelines or individual studies published in peer-reviewed literature. In some cases, only limited evidence was available; this is especially the case for formaldehyde, which uses a relative risk (RR) from a single study.

**Table 1:** Summary of health end points, exposure units, exposure–response relationships, and calculation methods.

Risk factor	Selected health end points	Population	Exposure estimate	Unit of exposure	Type of ERF	Point estimate of ERF (95% CI)^*a*^	References for ERF	Threshold^*b*^	Calculation method^*c*^
Benzene	Leukemia	All	Annual mean exposure	μg m^–3^	UR	6.00 × 10^–6^ (2.20 × 10^–6^, 7.80 × 10^–6^)	WHO 2000	0	2a
Dioxin	Total cancer incidence	All	Daily intake of adults	pg/kg/day	UR	1.00 × 10^–3^ (5.70 × 10^–4^, 5.10 × 10^–3^)	Leino et al. 2008; National Academy of Sciences 2004	0	2a
SHS	Trachea, bronchus, and lung cancers^*d*^	Adult nonsmokers	Percent of exposed	Yes/no	RR	1.21 (1.13, 1.30)	U.S. Surgeon General 2006	0	1a
SHS	Ischemic heart disease	Adult nonsmokers	Percent of exposed	Yes/no	RR	1.27 (1.19, 1.36)	U.S. Surgeon General 2006	0	1a
SHS	Asthma induction	Adult nonsmokers	Percent of exposed	Yes/no	RR	1.97 (1.19, 3.25)	Jaakkola et al. 2003	0	1a
SHS	Asthma induction	Children (< 14 yr)	Percent of exposed	Parental yes/no	RR	1.32 (1.24, 1.41)	Cal-EPA 2005	0	1a
SHS	Lower respiratory infections	Infants (< 2 yr)	Percent of exposed	Parental yes/no	RR	1.55 (1.42, 1.69)	U.S. Surgeon General 2006	0	1a
SHS	Otitis media	Toddlers (< 3 yr)	Percent of exposed	Parental yes/no	RR	1.38 (1.21, 1.56)	Cal-EPA 2005; Etzel et al. 1992	0	1a
Formaldehyde	Asthma aggravation (children) (morbidity only)	Toddlers (< 3 yr)	Annual mean residential indoor concentration	μg/m^–3^	RR	1.017 (1.004, 1.025)	Rumchev et al. 2002	100	1a
Lead	IQ loss	Children (< 5 yr)	Blood lead levels	μg/L	UR	0.051 (0.032, 0.07)	Lanphear et al. 2005	24	NA
Lead	Mild mental retardation (morbidity only)	Children (< 5 yr)	Blood lead levels	μg/L	DS^*e*^	Function^*f*^	—	24	2b
Lead	Hypertensive diseases (morbidity only)	Adults/all	Blood lead levels	μg/L	DS^*e*^	Function^*f*^	—	50	2b
Lead	Increased blood pressure	Adults/all	Blood lead levels	μg/L	UR	2.50 × 10^–2^ (1.70 × 10^–2^, 3.20 × 10^–2^)	Fewtrell et al. 2003; Schwartz 1995	50	NA
Road traffic noise	Severe sleep disturbance (morbidity only)	All	Exposure categories	*L*_night_ (dB)	UR	Function^*f*^	Miedema and Vos 2007; WHO 2009c	35	2b
Road traffic noise	Ischemic heart disease (mortality and morbidity)	All	Exposure categories	*L*_day_16hr (dB)	OR	Function^*f*^	Babisch 2006, 2008	55	1a
Railway traffic noise	Severe sleep disturbance (morbidity only)	All	Exposure categories	*L*_night_ (dB)	UR	Function^*f*^	Miedema and Vos 2007; WHO 2009c	35	2b
Aircraft noise	Severe sleep disturbance (morbidity only)	All	Exposure categories	*L*_night_ (dB)	UR	Function^*f*^	Miedema and Vos 2007; WHO 2009c	35	2b
Ozone	Total mortality (non-violent)	Adults (> 30 yr)	Ambient SOMO35 level	μg/m^–3^	RR	1.0003 (1.0001, 1.0004)	WHO 2006a	70	1a
Ozone	Minor restricted activity days (morbidity only)	Working age (18–64 yr)	Ambient SOMO35 level	μg/m^–3^	UR	0.0115 (0.0044, 0.02)	Hurley et al. 2005; WHO 2006b	70	2b
Ozone	Cough days, children (morbidity only)	Schoolchildren (5–14 yr)	Ambient SOMO35 level	μg/m^–3^	UR	0.093 (0.019, 0.22)	Hurley et al. 2005; WHO 2006b	70	2b
Ozone	LRS days in children (excluding cough) (morbidity only)	Schoolchildren (5–14 yr)	Ambient SOMO35 level	μg/m^–3^	UR	0.016 (–0.043, 0.08)	Hurley et al. 2005; WHO 2006b	70	2b
PM_2.5_	Cardiopulmonary disease (mortality and morbidity)	Adults (> 30 yr)	Population-weighted ambient level	μg/m^–3^	RR	1.0077 (1.0020, 1.0132)	Pope et al. 2002; WHO 2006a	0	1a
PM_2.5_	Lung cancer (mortality and morbidity)	Adults (> 30 yr)	Population-weighted ambient level	μg/m^–3^	RR	1.012 (1.004, 1.020)	Pope et al. 2002; WHO 2006a	0	1a
PM_2.5_	Chronic bronchitis (new cases) (mortality and morbidity)	Adults (> 27 yr)	Population-weighted ambient level	μg/m^–3^	UR	5.33 × 10^–5^ (1.70 × 10^–6^, 1.13 × 10^–4^)	Hurley et al. 2005; WHO 2006b	0	2b
PM_2.5_	Restricted activity days (morbidity only)	15–64 yr	Population-weighted ambient level	μg/m^–3^	UR	0.0902 (0.0792, 0.101)	Hurley et al. 2005; WHO 2006b	0	2b
Radon	Lung cancer (mortality and morbidity)	All	Residential mean level	Bq/m^–3^	RR	1.0016 (1.0005, 1.0031)	Darby et al. 2005, 2006	0	1a
Abbreviations: Cal-EPA, California Environmental Protection Agency; DS, distribution shift; ERF, exposure–response function; *L*_day_16hr, noise level for day and evening; LRS, lower respiratory symptoms; NA, not applicable; PM_2.5_, particulate matter ≤ 2.5 μm; RR, relative risk; SOMO35, sum of maximum 8-hr ozone levels > 35 plead (70 μg/m^3^); UR, unit risk; yr, years.^***a***^Exposure–response functions are all expressed per 1 unit of exposure. ^***b***^Above the threshold the health impacts are included in the estimates. ^***c***^Different types of calculation methods were applied, as described in “Methods.” ^***d***^The RR for spousal smoking is used as a proxy for any regular exposure (including at work). ^***e***^For lead, a shift in exposure distributions is linked to a unit risk approach. ^***f***^No point estimate can be given because the exposure–response function is given by a more complex function. This table is adapted from the full report (Hänninen and Knol 2011) with the permission of the copyright holder.									

The EBD was estimated only for exposures above defined thresholds, if any, using a comparative risk assessment method based on a counterfactual exposure distribution that would result in the lowest population risk. The feasibility of reaching the counterfactual exposure levels in practice was not considered.

*Estimation of the EBD.* Three different methods (methods 1a, 2a, or 2b) were used to estimate the EBD, depending on the type of exposure–response function estimate available for each exposure–outcome pair [either an RR based on environmental epidemiology, or a unit risk (UR) based on toxicological or occupational data], and on the availability of a WHO baseline burden of disease (BD) estimate (WHO 2009b) for the outcome. The method used for each exposure–outcome relation is listed in Table 1.

When a WHO BD was available for a given outcome, the EBD was estimated based on the population-attributable fraction (PAF) for that outcome in relation to each exposure of interest,

*EBD* = *PAF* × *BD*. [1]

Two methods (1a, 2a) were used to estimate the PAF, depending on the type of exposure–response function estimate available.

Method 1a. For exposure–outcome pairs with an RR estimate, the PAF is derived as (Rockhill et al. 1998)

*PAF* = [*p* × (*RR* –1)]/[*p* × (*RR* – 1) + 1], [2]

where *p* is the proportion of population exposed and *RR* is the relative risk at the level of exposure.

Method 2a. URs were used to estimate the PAF for exposure–outcome pairs without RR estimates available. URs, which are an estimate of the number of cases expected at a certain level of exposure, allow for direct estimation of the number of attributable cases (AC) from the exposure data:

*AC* = *E* × *UR* × *P*, [3]

where *E* is the exposure level, *UR* is the unit risk, and *P* is the size of the exposed population. The PAF is estimated from the AC as

*PAF* = *AC*/*I*, [4]

where *I* is the total incidence of the studied end point. The *EBD* is then estimated using Equation 1. This method will slightly overestimate the impact of the environmental exposure on mortality by including also nonfatal cases in AC, but allows for using standard WHO burden of disease data. The overestimation depends on the site of the cancer in question and is small for highly fatal cancers (e.g., lung cancer) but larger for less fatal cancers (e.g., childhood leukemia) and total cancers.

Method 2b. For outcomes without a WHO BD estimate available (e.g., severe sleep disturbance), the EBD was estimated as

*EBD* = *AC* × *DW* × *L*, [5]

where *AC* is the number of attributable cases (estimated using UR and Equation 3), *DW* is the disability weight characterizing the severity of the disease [ranging from 0 (perfect health) to 1 (death)], and *L* is the average number of years lived with disability (YLD) for morbidity effects, or years of life lost for mortality (YLL).

Results were calculated both using the WHO Global Burden of Disease 2004 (WHO 2008b) approach with age weighting and discounting (3%) and without age-weighing and discounting [as done in the Global Burden of Disease 2010 study (Lim et al. 2012)]. Additionally, as some of the health outcomes such as cancers have long incubation periods between exposure and clinical detection of the disease, these lag times were considered in the discounted model. However, in this review all results are presented without discounting and age-weighting. Discounting affects significantly the magnitude of the estimates in case of premature mortality and chronic conditions, up to a factor of 2. However, comparisons of the discounted and nondiscounted results showed that the ranking of exposures was not very sensitive to the choice of discounting and age-weighting or not. See the project report (Hänninen and Knol 2011) for a more comprehensive discussion on this.

*Selection of health end points*. Health end points and dose–response coefficients are summarized in Table 1.

Benzene effects were estimated for leukemia, including morbidity and mortality. Other proposed health end points were not included because they occur only at high exposure levels, typical of occupational settings. We used the exposure–response function as recommended by the WHO *Air Quality Guidelines for Europe* (WHO 2000).

The effect of exposure to dioxins and dioxin-like PCBs were estimated on cancer (all cancer types). The noncancer effects were not considered because of difficulties in estimating the exposure–response relationships and the other input parameters necessary for estimating DALYs; therefore, the estimates were calculated by first assuming all attributable cancer cases fatal during the first year after clinical detection and then using PAF from Equation 4 in method 2a. Leino et al. (2008) assumed a linear exposure–response relationship for excess cancers associated with dioxin intake. They estimated the health risk for toxicity equivalent intake assuming additivity of the toxicity of the different types of dioxins and all cancer cases to be lethal.

The EBoDE calculations used the Leino et al. (2008) approach, but the results have been corrected with an updated cancer slope factor 1 × 10^–3^ per pg/kg/day of dioxin intake of the U.S. Environmental Protection Agency (National Academy of Sciences 2004; U.S. Environmental Protection Agency 2003). The assumption that all cancers are lethal may lead to overestimation of the impacts.

Of the large number of health end points that SHS is associated with, we selected mortality and morbidity due to lung cancer and ischemic heart disease (IHD), morbidity due to onset of asthma (both in children and in adults), lower respiratory infections, and acute otitis media. For the other health end points mentioned above, strong evidence is available, but the necessary disease statistics were lacking. For the SHS-related burden of disease calculations, we have followed the recent WHO methods on the global estimation of disease burden from SHS (Öberg et al. 2011). The selected outcomes are being applied only to nonsmokers—to the nonsmoking disease burden. To that effect, the disease burden due to active smoking has been deduced from the total disease burden, by country [based on total disease burden and active smoking disease burden by country provided by WHO; update 2002 based on Ezzati and Lopez (2004)].

The development of asthma in toddlers was the only health end point included for formaldehyde (Rumchev et al. 2002). Sinonasal cancer, observed at occupational exposure levels, has been ruled out by WHO Air Quality Guidelines working groups, which have concluded that there is no epidemiological or toxicological evidence that formaldehyde would be associated with sinonasal cancer at levels < 1 mg/m^3^ (WHO 2000, 2010a). The WHO *Guidelines for Indoor Air Quality* (WHO 2010a) use eye irritation as the main health end point associated with formaldehyde; however, because of difficulties in estimating a burden of disease from irritation, this end point was not included in our calculations.

The estimates for lead include two end points that have been shown to be relevant at current exposure levels: mild mental retardation (due to IQ loss) and hypertensive disease (due to rise in systolic blood pressure). These associations exist at levels < 100 μg/L (Canfield et al. 2004; Carta et al. 2005; Walkowiak et al. 1998). Therefore, an extrapolation of the exposure–response curve to the range < 100 μg/L seems adequate. Lanphear et al. (2005) proposed a log-linear model for this curve.

Health end points associated with traffic noise included severe sleep disturbance and ischemic heart disease (IHD) (Babisch 2006, 2008; Miedema and Vos 2007). Hypertension and related heart disease due to aircraft noise was not considered because no clear review could be identified at that time. Nevertheless, because causal relationships are very likely and have been reported recently, this health effect may be considered in the future (Babisch and van Kamp 2009; van Kempen and Babisch 2012). For railway noise, no significant associations with hypertension and IHD could be identified either (Barregard et al. 2009). Effects on cognition and severe annoyance were excluded because these are difficult to quantify.

For ozone, as well as for PM, we followed the quantification approach as laid out in the Clean Air For Europe (CAFE) project and based on WHO European Centre for Environment and Health and CLTRAP (Convention on Long-Range Trans-Boundary Air Pollution) Task Force on Health consultations (Hurley et al. 2005). Health effects that are taken into consideration include total nonviolent mortality, minor restricted-activity days (MRADs), and cough and lower respiratory symptoms in children 5–14 years of age (WHO 2008a).

PM_2.5_ and PM_10_ (particulate matter with aerodynamic diameter ≤ 2.5 and ≤ 10 μm) both serve as indicators of a complex mixture of physically and chemically heterogeneous composition. The burden of disease related to both PM_10_ and PM_2.5_ exposures was calculated, but because of the overlap between these two indicators, in the aggregate results only the results for PM_2.5_ are presented. For PM_2.5_, we calculated the burden of disease for cardiopulmonary mortality, lung cancer mortality, total nonviolent mortality, chronic bronchitis and restricted-activity days [defined by Hurley et al. (2005)]. Because of the overlap between the different mortality end points, we report only cause-specific mortality in the aggregate results. For mortality, we used the RRs as provided by Pope et al. (2002; see also WHO 2006a, 2006b). For morbidity, RRs are based on the thorough review made for the CAFE estimates by Hurley et al. (2005) and WHO (2006b).

Radon effects are usually presented as additional cases of lung cancer at a certain exposure (i.e., UR model). To account for the interaction with smoking, however, an RR model seems more appropriate. We therefore calculated results using both a UR model and the RR model (methods 1a and 2a). The RR method 1a results are presented as the final results. The RR model, as suggested by the meta-analysis of Darby et al. (2005), assumes the lung cancer risk from radon to be linearly proportional to the radon exposure, but also to the background lung cancer rate caused by tobacco smoking and, to a lesser extent, by exposure to SHS and ambient air particulate matter and possibly some occupational exposures.

*Exposure data*. Calculations were carried out for the year 2004, the latest year for which exposure and health data were sufficiently available for the studied countries. Exposure data were preferably collected from internationally harmonized sources (Table 2), but in the case of benzene, dioxins, formaldehyde, and lead, complementary national data were needed. Population average data were used for all age groups when age group–specific data were lacking. More details are available in the project report (Hänninen and Knol 2011, Chapter 3).

**Table 2:** Sources for exposure data.

Stressor	Year(s) of original exposure data	Assumptions for trends estimation to 2004	Exposure data sources
Benzene	2004	National trend estimates when applicable	AirBase (2009) data for outdoor levels in 2004; national studies for indoors^*a*^
Dioxins	1997–2006	No trend assumed	National data for intake^*a*^
Secondhand smoke	2008	Available data fitted with power functions for trends	National^*a*^ and international survey data for exposures between 1990 and 2008 used for modeling 2004 data; EC 2009
Formaldehyde	1990–2005	No trend assumed	National indoor concentration data^*a*^
Lead	1990–2005	National trend estimates	National blood lead level data^*a*^
Traffic noise	2007^*b*^	No trend assumed	EC Environmental Noise Directive data
Ozone	2005	No trend assumed	ECT/ACC spatial model based on AirBase (2009) observations and air quality maps
Particulate matter	2005	No trend assumed	ECT/ACC spatial model based on AirBase (2009) observations and air quality maps
Radon	Up to 2005	No trend assumed	RadonMapping project (http://radonmapping.jrc.ec.europa.eu) and the UNSCEAR 2000 Report
For more details, see Hänninen and Knol (2011). Abbreviations: EC, European Community; ECT/ACC, European Topic Centre on Air Pollution and Climate Change Mitigation; UNSCEAR, United Nations Scientific Committee on the Effects of Atomic Radiation. ^***a***^References to sources of national exposure data are presented in the Supplemental Material, pp. 14–15. ^***b***^Target year of Environmental Noise Directive data was set as 2007. The actual collected data contains subsets of data from various years.			

*Uncertainty estimation and alternative analyses*. Many factors can contribute to uncertainty in EBD estimates (Knol et al. 2009), including the selection of risk factors and health effects, exposure data, exposure–response functions, and methodological choices. Some of these sources of uncertainty can be handled quantitatively, whereas others can only be described qualitatively. For the quantitative part, we have estimated statistical confidence intervals based on the uncertainty ranges of the exposure–response functions. In addition, we carried out several alternative analyses to explore the robustness and sensitivity of our results. We tested the effect of lag times from exposure to the onset of the disease and compared PM and ozone results to those obtained by using life tables, and we used a variety of different assumptions for our input data and models in selected scenarios. Details of these analyses are available in the project report (Hänninen and Knol 2011, Chapter 5).

For the qualitative part, we used expert judgment (provided by the thematic experts participating in the project) to evaluate the knowledge base to support the claim of causality between exposure and effect and other main factors affecting the model uncertainty.

## Results

Unless otherwise specified, all DALYS are presented as population-weighted nondiscounted and non-age-weighted annual averages. European results are calculated as weighted averages accounting for the size of population in each participating country.

**Figure 1:**
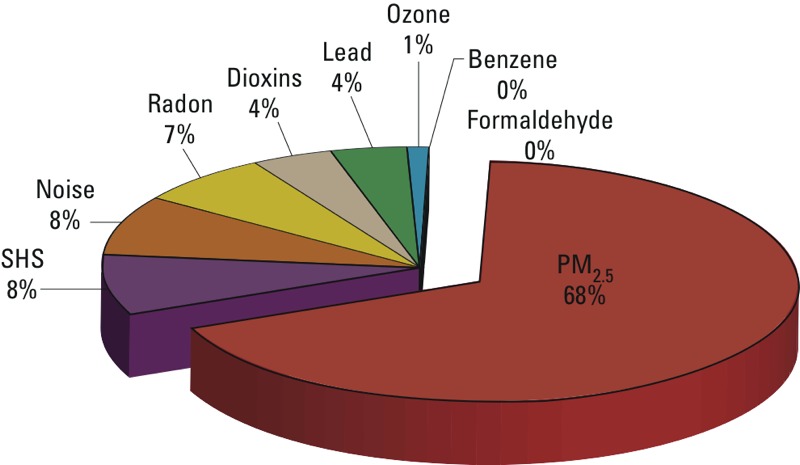
Relative contributions of the nine targeted risk factors to the estimated burden of disease attributed to these risk factors, averaged over the six participating countries. The figure is adapted from Hänninen and Knol (2011) with permission from the copyright holders.

**Figure 2:**
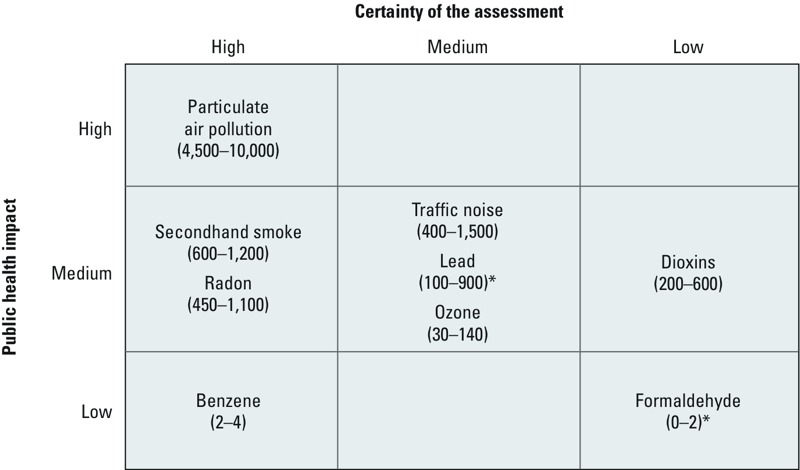
Ranges for the estimated contributions of the selected environmental risk factors to the burden of disease (DALYs per million people) as population-weighted averages over the six participating countries. Numerical values indicate nondiscounted DALYs per million people in the six participating countries.The figure is adapted from Hänninen and Knol (2011) with permission from the copyright holders. *A numerical model was used to estimate threshold exceedances.

*Overall results*. The central EBD estimates per environmental risk factor ranged from 2 to 10,000 DALYs per million people in the six participating countries (Belgium, Finland, France, Germany, Italy, and the Netherlands). The relative population-weighted contributions of the risk factors are shown in Figure 1, dominated by PM (68%), followed by SHS and traffic noise (8% each) and radon (7%). The estimated EBD was clearly dominated by PM_2.5_, which accounted for about 4,500–10,000 DALYs per million people, followed by SHS (600–1,200), radon (450–1,100), and traffic noise (400–1,500) (Figure 2). Estimates for lead (100–900), ozone (30–140), and dioxins (200–600) were classified to have medium public health impacts. Benzene (2–4) and formaldehyde (< 2) had relatively the lowest public health impacts. Ranking orders varied between countries. Figure 2 shows the estimated EBD and the quantitative ranges of the estimates between the six participating countries. More elaborate expert judgment of overall uncertainties is presented in the full report (Hänninen and Knol 2011), where the statistical uncertainty of the exposure–response functions is combined with the estimated level of certainty of the underlying knowledge on causality.

For six risk factors the public health impacts are dominated by either morbidity (formaldehyde, lead, and traffic noise) or mortality (benzene, dioxins, and radon). The selection of health end points may be partly responsible for this finding. In total, the selected risk factors are associated with 1.6 million YLL in the participating countries, or 6,900 YLL per million inhabitants.

Specific estimates for health end points ranged from 0.1 to 4,600 DALYs per million people, with the highest impacts for cardiopulmonary mortality, lung cancer mortality, and chronic obstructive pulmonary disease related to PM_2.5_ exposure (4,600, 1,500, and 1,200 DALYs per million people, respectively). These were followed by lung cancer (radon; 830 DALYs per million), severe sleep disturbance (traffic noise; 720 DALYs per million), and ischemic heart disease (SHS; 680 DALYs per million).

The total national burden of disease was estimated to range from 112,000 DALYs per million people in Italy to 132,000 in Finland (WHO 2009b). The nine investigated risk factors contributed 3.3–6.9% to the total estimated burden of disease, with the highest contribution in Italy and the lowest in Finland. In the intermediate countries the contribution of EBD to the total burden of disease was 6.3% in Belgium, 4.4% in France, 5.4% in Germany, and 5.6% in the Netherlands. The risk factor–specific DALYs per country are presented by Hänninen and Knol (2011, pp. 87–93) and in the Supplemental Material, Figures S1 and S2.

*Results and uncertainties by risk factor*. Particulate matter. PM_2.5_ accounted for 68% of the total estimated EBD, making it the most significant environmental risk factor in our analysis (Figure 1). This is in line with results of similar assessments (de Hollander et al. 1999; Logue et al. 2012; Prüss-Üstün et al. 2011). In the six participating countries, PM_2.5_ is estimated to cause 1.8 million DALYs annually and 1.3 million YLL (i.e., premature mortality only). Overall, 73% of the health impacts due to PM_2.5_ exposure were estimated to be attributed to mortality. The estimated PM_2.5_ impact ranged from 4,600 per million people in Finland and France to 10,500 DALYs in Belgium.

Main uncertainties relate to the exposure–response functions and the potential of double-counting of morbidity effects by combining restricted activity days and lower respiratory symptom days. Overall, PM is the most thoroughly reviewed risk factor included in this study.

SHS. The EBD related to SHS was estimated to account for 600–1,200 DALYs per million people. This is well in line with a large recent EBD assessment (Öberg et al. 2011) that estimated about 610 DALYs per million people in Western Europe.

Main uncertainties in our estimates relate to the difference between survey-based exposure measurements, matching between measured exposures and RRs, and the various assumptions made in applying the method (e.g., assuming that active smokers are not susceptible to SHS). Nonetheless, most evidence for SHS-related impacts is fairly consistent, and estimates of the EBD are considered relatively stable.

Estimated EBD from SHS is remarkably low in France (550 nondiscounted DALYs/million) and high in Germany (1,200), where exposure levels and baseline prevalence of the relevant diseases are higher.

Radon. Exposure to radon was estimated to cause 450–1,100 DALYs per million people. The radon-related EBD is the highest in France (1,100 nondiscounted DALYs/million) and Belgium (1,100), and lowest in the Netherlands (450). These differences are caused mainly by differences in geologically driven uranium concentrations in the soil, use of different building materials, and differences in national mitigation measures.

Traffic noise. Because so many people are exposed to traffic noise (including road, rail, and air traffic), the total estimated EBD associated with this exposure is substantial (400–1,500 DALYs/million), despite the relatively small disability weights for severe sleep disturbance (0.07). DALYs range from 370 per million people in less densely populated Finland up to 1,480 DALYs per million people in France. The exposure data, which were derived from the Environmental Noise Directive (2002) reporting from 2007, cover only agglomerations with > 250,000 inhabitants and roads outside these agglomerations with > 6 million vehicles per year, railroads with > 60,000 passages per year and airports with > 50,000 flights per year. Therefore, the results are probably an underestimation of the total burden in a country. In addition, only exposure levels above *L*_night_ (8-hr nighttime noise level) 50 dB [*L*_den_ (combined day-evening-night noise level) 55 dB] were available, so health impacts could not be estimated for lower exposure levels.

Dioxins, furans, and dioxin-like PCBs. The EBD related to dioxins in food was estimated to range from 240 to 580 DALYs per million people. Uncertainties are large: Effects of dioxins cannot easily be distinguished from other chemicals; low-dose effects are difficult to assess; and thresholds for effects are mostly unknown. Our estimates are based on the simplification of assuming that each cancer case was fatal during the first year when calculating the PAF using method 2a. Noncancer effects were not considered because of a lack of dose–response functions or quantifiable health end points. The PAF estimation method used could lead to a slight overestimation of dioxin effects due to counting nonfatal cases in the body count. On the other hand, ignoring noncancer effects could lead to an underestimation. We were not able to quantify these counteracting uncertainties. The EBD of dioxin exposure varies because of differences in diets and food contamination, and the different methods used to evaluate daily intake.

Lead. Lead was estimated to contribute to 100–900 DALYs per million people. The underlying exposure data had limited population representativeness and were based partly on older data supplemented with trend estimations. Other uncertainties relate to unavailability of exposure–response functions over the complete exposure spectrum as well as the aggregation of effects. Lead exposures were the highest in Italy. One reason for this may be that the exposures were measured in adults only. In the Netherlands, in contrast, the sample included children 1–6 years of age. Because lead accumulates in the body over the years, this is probably the most important reason why lead-related EBD is relatively low in the Netherlands (220 nondiscounted DALYs/million) and relatively high in Italy (950). More consistent human biomonitoring data are needed for lead.

Ozone. The acute impacts of tropospheric ozone on public health ranged from 30 to 140 DALYs per million people. Uncertainties in the calculations relate, among other issues, to the estimated YLL due to mortality and chronic effects. Estimated ozone impacts were highest in the Mediterranean countries, represented here by Italy (140 nondiscounted DALYs/million). Levels in the Netherlands were the lowest (34), probably because of meteorological factors and relatively high levels of nitrogen oxide.

Benzene. The EBD of benzene in air was estimated to be < 5 DALYs per million people. Representativeness and comparability of exposure data were estimated to be the largest source of uncertainty.

Formaldehyde. The EBD related to formaldehyde in air was estimated as < 2 DALYs per million people. Formaldehyde levels in Finland are higher than in many other developed countries due to the types of construction materials used and the relatively tightly sealed buildings.

Main uncertainties related to the difficulties in selection of end points, thresholds, and very limited epidemiological data at prevailing exposure levels. We applied a threshold of 100 μg/m^3^ (WHO 2000, 2010a), which is exceeded very rarely in Europe.

## Discussion

*Policy relevance*. EBD estimates are aimed to support efficient policy development and resource allocation. International comparisons over a range of environmental risk factors, as presented in this study, form a valuable basis for prioritizing among environmental policies and for international benchmarking. International comparisons can also be a strong incentive for national policy development. Preliminary results of this study were greatly appreciated when presented at the fifth Ministerial Conference on Environment and Health in Parma in 2010 (WHO 2010b). Based on our results, PM is an obvious candidate that requires further reduction, whereas dioxins and formaldehyde seem to be less relevant from a population-wide EBD perspective. However, for these risk factors, policy action also may be required, for example, for specific susceptible groups. Our approach does not allow for estimating health impacts in specific population groups, such as highly exposed (e.g., occupational exposures) or other susceptible groups (gender, age, genetic predisposition). Such information is needed when developing specific policy measures and considering environmental equity, feasibility of policy measures, developing accountability studies, and evaluating health benefits, wellbeing, risk perception, and associated uncertainties.

Interpretation of the presented EBD estimates in the context of risk management and policy development requires care. Besides the inherent uncertainties, the EBD as calculated here cannot be directly interpreted as the total reduction potential. Some health impacts may always remain because of background concentrations from natural sources and practical limitations in removing anthropogenic pollution. Using expert judgment, Prüss-Üstün and Corvalán (2006) estimated the EBD related to modifiable environmental factors, which may be more relevant from a policy effectiveness perspective. As future research, it would be interesting to investigate the actual use and effect of EBD studies on national or international agenda setting, policy development, and policy evaluation.

*Uncertainties and limitations*. Because of the large number of data and knowledge needed for EBD calculations, many sources of uncertainties affect the results (Knol et al. 2009). Besides the parametric uncertainties, for which we have calculated numerical uncertainty ranges, we carried out a number of quantitative sensitivity analyses for model uncertainties, and also used expert judgments to provide a qualitative estimate of the knowledge base underlying the claims for causality.

Overall, we believe that the six country averages are likely to provide reasonable estimates of the magnitude of the environmental burden of disease in Western Europe, and that uncertainties will not affect the rank ordering of the estimated impacts of the risk factors, though estimated impacts of SHS, radon, and traffic noise do overlap. However, generalizability to other countries is limited by risk factor–specific issues. For example, radon exposures are highly variable, and the differences in exposure levels cannot be generalized.

The numerical uncertainty ranges presented here were based solely on uncertainty in the exposure–response functions. The evaluation of the knowledge base on causality, based on expert judgment, was considered to have the highest reliability for PM_2.5_, SHS, radon, and benzene. Medium uncertainties were identified for traffic noise, lead, and ozone, whereas dioxins and formaldehyde were considered most uncertain. Nonconclusive sensitivity analyses suggest that our overall ranking of risk factors is relatively robust against identified main sources of model uncertainties. Baseline comparison with other data-driven EBD studies (e.g., de Hollander et al. 1999; Logue et al. 2012; OECD 2001) confirms relative robustness of the overall ranking and order of magnitude of the estimates, despite methodological differences and variation in baseline assumptions.

We included in our EBD estimates only impacts for which sufficient evidence and quantitative data were available. The availability of data and evidence was evaluated by the experts who participated in the study. Health effects that are suspected but not sufficiently researched or monitored, as well as health effects that fall outside the scope of the ICD-10 coding system, were not included. Expert elicitation, such as that used by Prüss-Üstün and Corvalán (2006) and structured by Knol et al. (2010), may be useful in filling some of these gaps.

The exposure data we used had varying degrees of temporal, population, and geographical coverage. Exposure data collected with standardized methods over all the participating countries were available for PM_2.5_ and ozone from the European air quality monitoring system (AirBase 2009) (see Table 2). Radon, SHS, benzene, and dioxins had reasonably comparable data. Radon exposures are monitored by national programs and have been extensively reviewed by international research groups (Darby et al. 2005). The SHS exposure questionnaire was conducted in all European countries (European Community 2009). Also, dioxins have been extensively reviewed, even though there were differences in data availability between the countries. Traffic noise data collection is well defined in the European Noise Directive (2002), but the comparability of the data available from the first phase of this directive had not yet reached these standards at the time of collecting the present data. The lowest comparability of exposure data was found for lead and formaldehyde data, for which the assessments were based only on studies with no international standardization in population sampling, seasonal variability, and temporal trend estimation. This can be considered surprising. Lead has been a very important pollutant in the past, and policy evaluation and follow-up would require comparable and representative exposure data. In several countries, lead exposure levels have been in strong decrease over recent years, as documented for instance for Italy (Alimonti et al. 2011).

International monitoring standards and procedures could strengthen data quality and improve comparability. The current lack of harmonized environmental exposure data is one of the things that hinders comparable EBD assessments and policy evaluation.

*Discounting, age-weighting, and lag times*. When calculating DALYs, it is optional to discount or age-weight the results. Discounting is based on the assumption that future years of healthy life are considered less valuable than years of healthy life at the present time. Non-uniform age weighting means that a year lived at a younger or older ages is given a lower value than a year lived by a young adult. The use of both discount rates and age-weighting has been debated (Anand and Hanson 1997; Arnesen and Nord 1999; Schneider 2001). Discounting leads to lower valuation of impacts that occur later or last longer, in comparison with immediate effects. This is not favorable for children and future generations, and it devalues preventive measures. The use of age weights is also controversial because it values the lives of children and elderly less than other lives. Therefore, in this study we have chosen not to discount or age-weight our main results. The recent Global Burden of Disease 2010 study (Lim et al. 2012), coordinated by Institute of Health Metrics, also rejected discounting and age-weighting (Lim et al. 2012).

We performed additional analyses to explore the effects of discounting and age-weighting (Hänninen and Knol 2011, p. 70). The overall ranking of the risk factors was more or less stable against the alternative discounting procedures. However, the absolute magnitude of the estimated impacts was reduced to one-third of the nondiscounted value by discounting and age-weighting for diseases associated with substantial premature mortality and chronic diseases, for instance in case of lung cancer associated with SHS, PM_2.5_, and radon. In other contexts, such as debates over nuclear energy, the health of future generations is often given priority over benefits of the current economy. Moreover, children’s health has been set as a priority in the European Environmental Health Action Plan (WHO 2010b). This contrasts with the consequences of discounting and age-weighting, which downscale health impacts in children.

## Conclusions

EBD was estimated for nine environmental risk factors (benzene, dioxins, formaldehyde, SHS, lead, traffic noise, PM_2.5_, ozone, and radon) in six countries. The highest overall public health impact was estimated for ambient fine particles (PM_2.5_; annually 4,500–10,000 nondiscounted DALYs/million in the six participating countries) followed by SHS (600–1,200), traffic noise (400–1,500), and radon (450–1,100). Medium impacts were estimated for lead, dioxins, and ozone. Lowest impacts were estimated for benzene and formaldehyde. The relative ranking of the risk factors was relatively robust under the uncertainties examined.

EBD assessment is useful for setting research and risk management priorities from the point of view of public health benefits and resource allocation. This may include both the identification of susceptible population groups and health-based evaluation of the efficiency of potential benefits from exposure reduction policies. Further development of methods to address additional risks and health outcomes would allow a more complete account of health impacts caused by environmental risks. International exposure monitoring standards and activities would improve data availability, strengthen data quality, and improve comparability.

## Supplemental Material

(655 KB) PDF
