# Supplementary material for: Environmental Burden of Disease in Europe: Assessing Nine Risk Factors in Six Countries
Source: Environ Health Perspect. 2014 Feb 28;122(5):439–46. doi: 10.1289/ehp.1206154 (PMC4014759; doi:10.1289/ehp.1206154)
Supplement: (655 KB) PDF [file ehp.1206154.s001.pdf]

Supplemental Material

Environmental Burden of Disease in Europe: Assessing Nine Risk Factors in Six Countries

Otto Hänninen, Anne B Knol, Matti Jantunen, Tek-Ang Lim, André Conrad, Marianne Rappolder, Paolo Carrer, Anna-Clara Fanetti, Rokho Kim, Jurgen Buekers, Rudi Torfs, Ivano Iavarone, Thomas Classen, Claudia Hornberg, Odile C.L. Mekel, and the EBoDE Working Group

Table of Contents | Page

Results by country 3
Calculation parameters and uncertainties 3
Table S1. Input parameters for calculating the burden of disease (method 2b). 4
Figure S1. Undiscounted, un-age-weighted DALYs per million people per country. 5
Figure S2. Environmental fraction attributable to the studied nine stressors of the total burden of disease in the participating countries (discounted age-weighted DALYs per million people). 8
Table S2. Identified sources of uncertainty in the calculations. 9
Table S3. Parameters of alternative calculations. Bold values indicate the varied parameters in comparison to the baseline. 12
References to national exposure data sources 14
Benzene 14
Belgium 14
Finland 14
France 15
Germany 15
Italy 15
The Netherlands 15
Dioxins (including furans and dioxin-like PCBs) 16
Belgium 16
Finland 16
France 16
Germany 16
Italy 17
the Netherlands 17

Second hand smoke 17
International sources *17*
Belgium *17*
Finland *17*
France *18*
Germany *18*
Italy *18*
The Netherlands *19*
Formaldehyde 19
Belgium *19*
Finland *19*
France *19*
Germany *19*
Italy *19*
The Netherlands *20*
Lead 20
Belgium *20*
Finland *20*
France *20*
Germany *21*
Italy *21*
The Netherlands *21*

Results by country

This section provides supplemental material for the environmental burden of disease analysis conducted for nine stressors in six European countries.

Figure S1 and **Figure S2** show national results by country. **Figure S1** shows results by stressor. **Figure S2** shows the overall environmental burden of disease results by country compared with the total national burden of disease.

Calculation parameters and uncertainties

Table S1 shows the input parameters for calculating the burden of disease for exposure-response associations estimated using Method 2b (unit risk and bottom up calculation of burden of disease based on duration and disability weight of cases).

Table S2 presents the identified uncertainties associated with the estimates. Numerical calculations were conducted to those factors presented in **Table S3**. The results of the uncertainty calculations are available in the project report (Hänninen & Knoll, 2011).

Table S1. Input parameters for calculating the burden of disease (method 2b).

**Stressor**	**Health endpoint**	**Disability Weight**	**Duration (yrs)**
Lead	Mild mental retardation	0.36	77.6
Lead	Hypertensive disease	0.2	3.6
Road traffic noise	High sleep disturbance (HSD)	0.07 [a]	1
Railway noise	High sleep disturbance (HSD)	0.07 [a]	1
Aircraft noise	High sleep disturbance (HSD)	0.07 [a]	1
Ozone	Minor restricted activity days	0.07 [b]	0.00274 (= 1 day)
Ozone	Cough days, children	0.07 [b]	0.00274 (= 1 day)
Ozone	LRS days in children (excl cough)	0.099 [c]	0.00274 (= 1 day)
$PM_{2.5}$	Restricted activity days (RAD)	0.099 [c]	0.00274 (= 1 day)

[a]Disability weight proposed by the WHO working group for noise impact assessment (confidence intervals 0.04-0.09). [b]Disability weight for pharyngitis. [c]Disability weight for lower respiratory infections (chronic sequelae).

This table is reproduced with permission from the copyright holder of Hänninen and Knol, 2011.

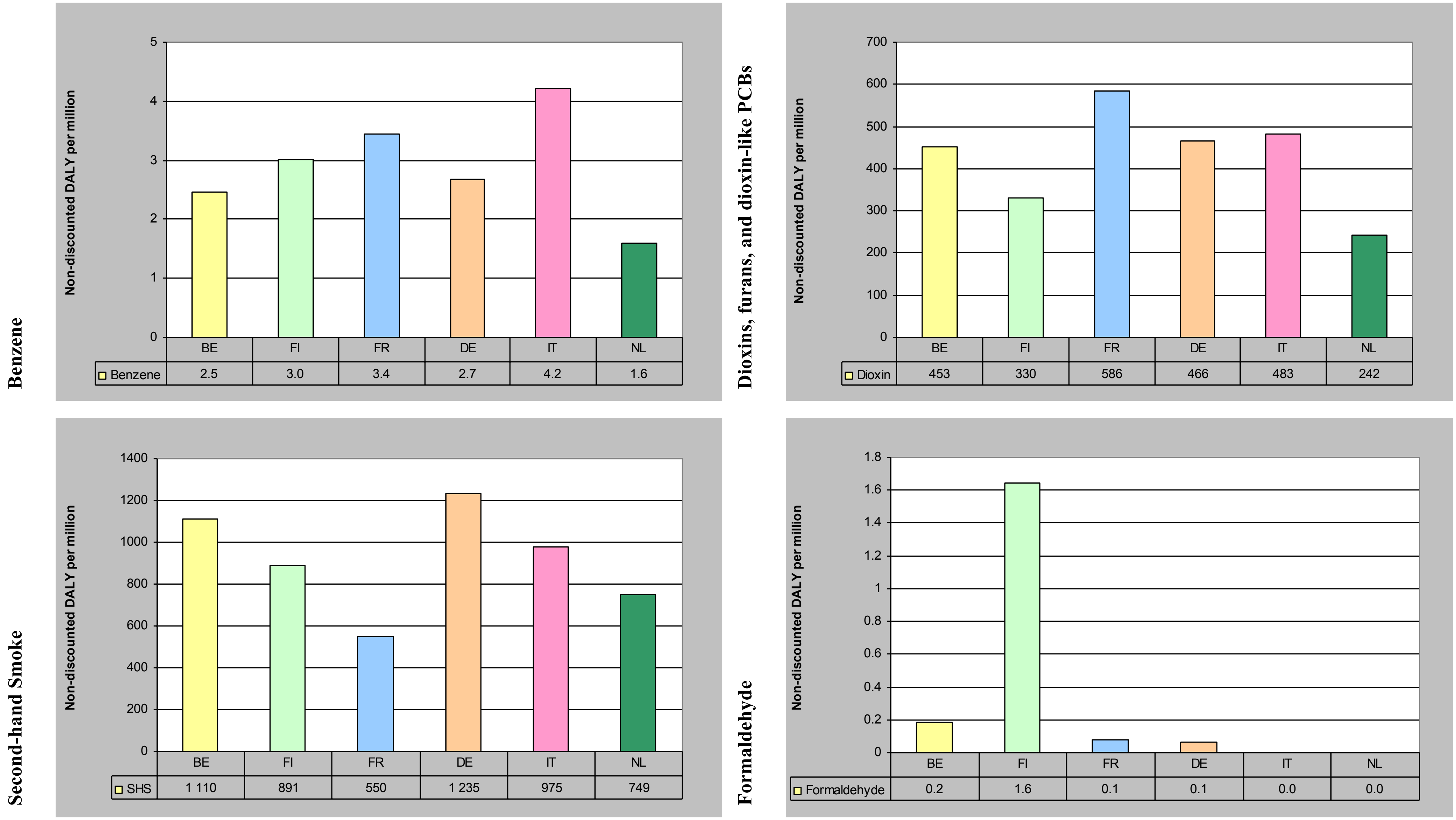

Figure S1. Undiscounted, un-age-weighted DALYs per million people per country. This figure is reproduced with permission from the copyright holder of Hänninen and Knol, 2011.

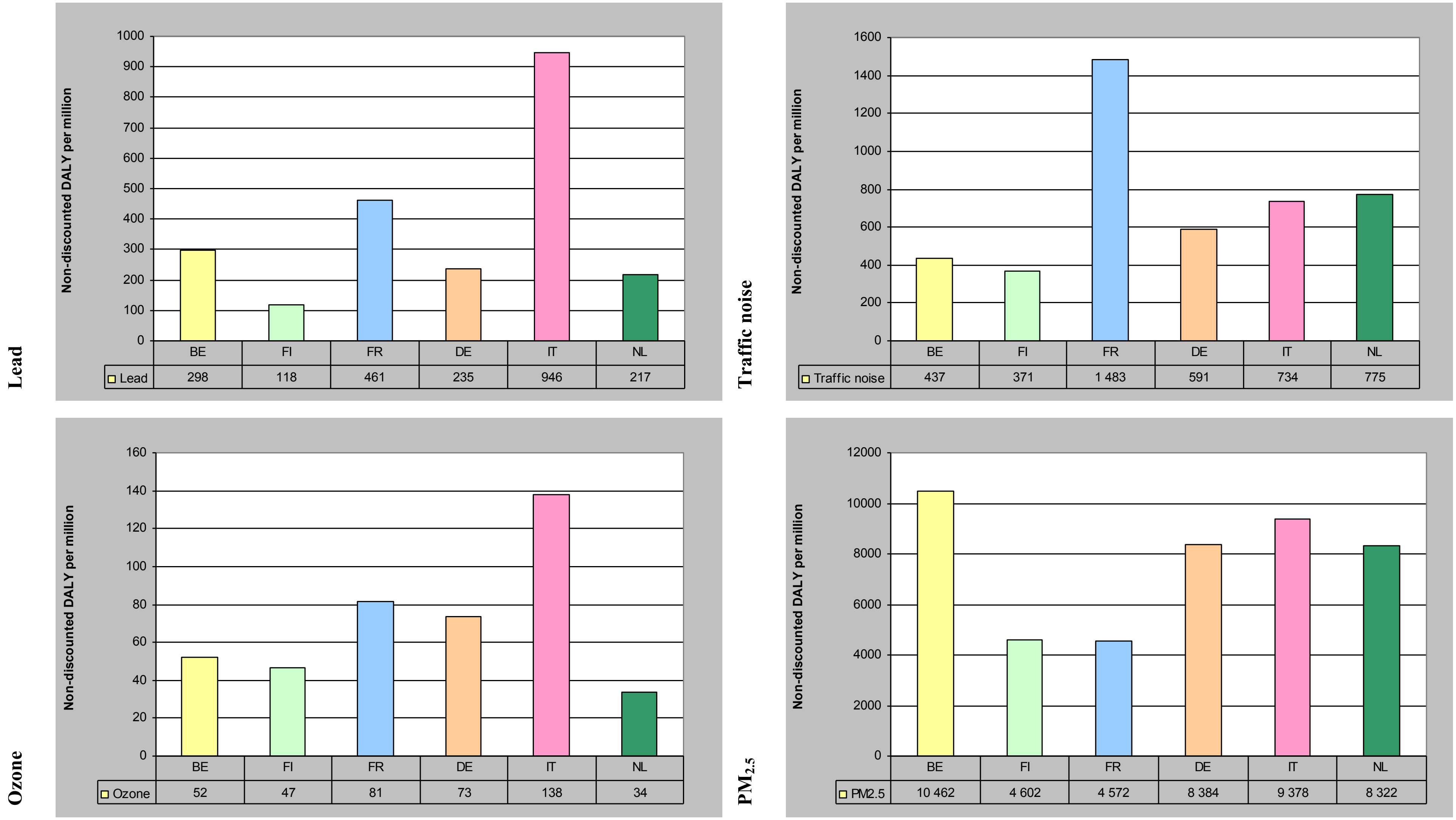

Figure 1. (continued).

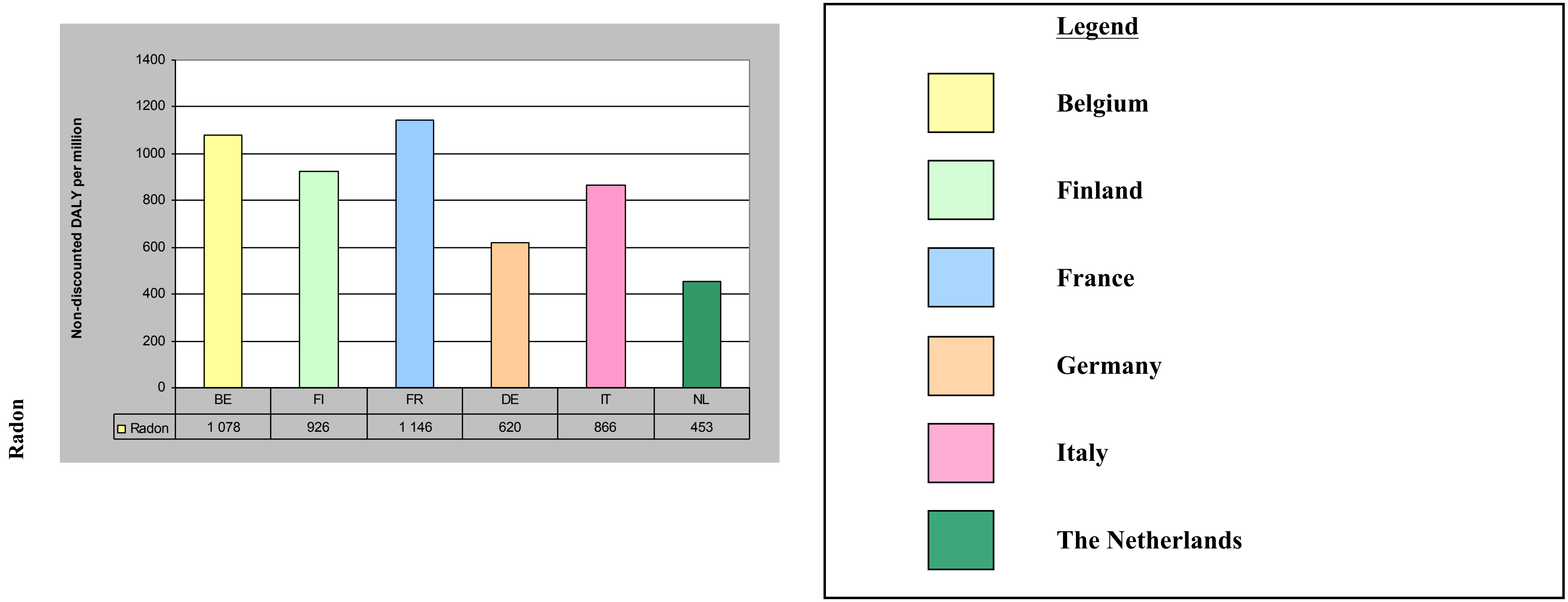

Figure 1. (continued).

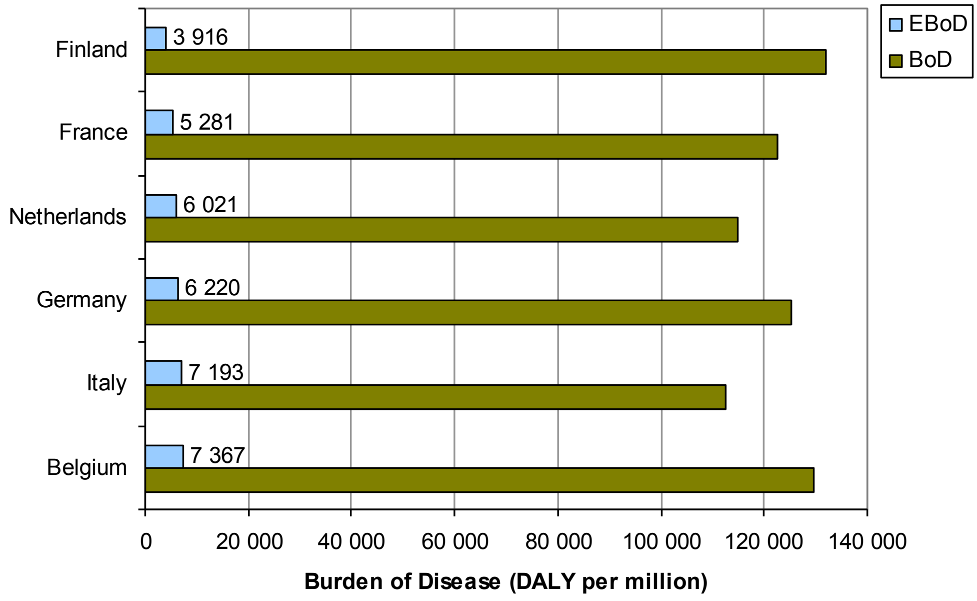

Figure S2. Environmental fraction attributable to the studied nine stressors (in blue) of the total burden of disease in the participating countries (discounted age-weighted DALYs per million people). This figure is reproduced with permission from the copyright holder of Hänninen and Knol, 2011.

Table S2. Identified sources of uncertainty in the calculations.

Risk factor	Selection of health endpoints that are not included (but for which supporting evidence exists) and related assumptions	Exposure data	Exposure response function	Calculation method	Level of overall uncertainty [a]	Likely over- or underestimation [b]
Benzene	Anaemia; genotoxicity; other blood cancers than leukaemia; leukaemia morbidity; effects on the immune, endocrine and nervous system; acute effects. All cases of leukaemia assumed to be fatal.	- Population representativity varies<br>- Differences in number of dwellings<br>- Different types of measurements (indoor/outdoor; in – or excluding SHS, etc)<br>- Sampling times differ	- No specific relationships for children used (i.e. same UR used for all ages)	UR method of calculating PAF results in overestimation because all cases are assumed to be fatal.	*	Underestimation due to excluded health endpoints, but overestimation due to UR method
Dioxins (plus furans and PCBs)	Effects on the immune, endocrine, reproductive and nervous system; tooth and bone defects. All cases of cancer assumed to be fatal.	- Indirect exposure metrics<br>- Different measurement methods<br>- Daily intake of food depends on age, body weight and eating habits<br>- Exposure varies within countries (from region to region)	- Uncertain cancer slope factor<br>- Assumed additivity of the toxicity of different types	UR method of calculating PAF results in overestimation because all cases are assumed to be fatal.	***	Underestimation of non cancer effects, Overestimation of cancer effects (all lethal)
Second hand smoke	Sudden infant death syndrome; low birth weight; reduced pulmonary function among children; acute irritant symptoms. Odds ratios used as RR estimates	- Data from different years and consequent temporal interpolation<br>- Differing definitions of exposures<br>- Data gaps for some countries	- ERF from earlier decades when questionnaire responses may have been less sensitive	Various assumptions made, e.g. smokers are not susceptible to SHS	*	Underestimation due to excluded endpoints<br><br>Potential overestimation due to increased questionnaire sensitivity
Formaldehyde	Acute symptoms; nasopharyngeal and sinonasal cancers. Original data collected from <3 yr olds; potential effects at older ages not accounted for	- Data from different years<br>- Population representativity varies<br>- For some countries limited national coverage<br>- Limitation in technique to detect peak exposures	- Shape of ERF<br>- Threshold level<br>- Partly inconclusive evidence for the endpoint	- Simulation of threshold exceedances<br>- Selection of age groups	***	Underestimation, mainly due to exclusion of ≥ 3year olds but also not accounting for eye irritation

Risk factor	Selection of health endpoints that are not included (but for which supporting evidence exists) and related assumptions	Exposure data	Exposure response function	Calculation method	Level of overall uncertainty [a]	Likely over- or underestimation [b]
Lead	Other cardiovascular diseases than hypertensive disease; kidney damage; miscarriages; other effects of the nervous system; declined fertility; alterations in growth and endocrine function; behavioural disruptions; hearing-threshold changes; hyperkinetic syndrome; lung and stomach cancers. MMR: proxy for all lost IQ points	- Differences in study year<br>- Differences in studied age group<br>- Incomplete data, temporal extrapolation and poorly known exposure trends	- Threshold level<br>- Shape of ERF	- Evidence limited at prevailing low exposure levels<br>- Estimation of threshold exceedances	**	Underestimation due to excluded end-points
Transport noise	Annoyance; cognitive impairment, tinnitus	- Small proportion of target population is covered<br>- Conversion between different noise metrics<br>- Different samples<br>- Different data estimation years		- Disability weight for sleep disturbance is quite high<br>- MI vs IHD	**	Underestimation due to uncovered populations, low exposures, endpoints and noise sources
Ozone	Possible long-term effects	- Spatial interpolation<br>- Impact of urban areas		YLL not known	**	Overestimation (YLL set to 12 months)
Particulate matter	Morbidity outcomes evaluated using the CAFE simplifications	- total PM (not just anthropogenic emissions)	- Potential threshold level	- Unit risk simplifications for morbidity outcomes	*	No substantial error expected or overestimation due to inclusion of natural background
Radon	none	- possible oversampling of geographical regions known problematic			*	No substantial error expected

[a]Estimated level of overall uncertainty in burden of disease estimates for specific stressor (authors' judgment)

* relatively low level

** medium level

*** relatively high level

This level may deviate from the level of evidence as presented in Figure 4-1 of Hanninen and Knol (2011), which provides an estimate of the certainty of the underlying knowledge about causality.

[b]Authors' judgment about whether results are likely to over- or underestimate the true EBD, given the uncertainties.

This table is adapted with permission from the copyright holder of Hänninen and Knol, 2011.

Table S3. Parameters of alternative calculations. Bold values indicate the varied parameters in comparison to the baseline.

Stressor	Health endpoint(s)	Population	Exposure estimate	Unit of exposure	Type of ERF	Point estimate of ERF [a]	Reference(s) for ERF	Threshold	Calculation method [b]
Formaldehyde	Asthma aggravation (children)	Children (< 3 yr)	Mean residential indoor concentration	µg m^{-3}	RR	1.017	Rumchev et al. 2002	**40, 60**	1A
Formaldehyde	**Nasopharyngeal cancer**	**All**	Mean residential indoor concentration	µg m^{-3}	**UR**	**1.30×10^{-5}**	**Kerns et al. 1983**	0	2B
Lead	**Hypertensive disease**	Adults	Blood lead level	µg/l	**UR**	**2.50×10^{-2}**	**Fewtrell et al. 2003**	50	2B
Lead	**Ischemic heart disease, cerebrovascular disease, other cardiac diseases**	Adults	Blood lead level	µg/l	**RR**	**N.A.** (Age- and gender calculations conducted using a separate WHO model)	**Fewtrell et al. 2003**	50	1A
Transport noise	**Myocardial infarction**	All	Persons exposed to predefined exposure categories	Lday16h (dB)	OR	function	Babisch 2006	55	1A
Transport noise	**Ischemic heart disease**	All	Persons exposed to predefined exposure categories	Lday16h (dB)	OR	function	Babisch 2006	55	1A
$PM_{2.5}$	Cardiopulmonary diseases	Adults (> 30 yr)	Population weighted ambient level	µg m^{-3}	RR	1.0077	Pope et al. 2002, WHO 2006a	**2, 4**	1A
$PM_{2.5}$	Lung cancers	Adults (> 30 yr)	Population weighted ambient level	µg m^{-3}	RR	1.012	Pope et al. 2002, WHO 2006a	**2, 4**	1A
$PM_{2.5}$	**Total mortality (non-violent)**	**Adults (> 30 yr)**	Population weighted ambient level	µg m^{-3}	**RR**	**1.0058**	**Pope et al. 2002, WHO 2006b**	0	1A
PM_{10}	**LRS symptoms days, children**	**School children (5-14 yr)**	Population weighted ambient level	µg m^{-3}	**UR**	**0.186**	**Hurley et al. 2005, WHO 2006b**	0	1A

Stressor	Health endpoint(s)	Population	Exposure estimate	Unit of exposure	Type of ERF	Point estimate of ERF [a]	Reference(s) for ERF	Threshold	Calculation method [b]
PM_{10}	**LRS symptom days, adults**	**> 15 yr with chronic LRS**	Population weighted ambient level	$\mu g\ m^{-3}$	**UR**	**0.13**	**Hurley et al. 2005, WHO 2006b**	0	1A
Radon	Lung cancer (mortality)	All	Residential mean level	$Bq\ m^{-3}$	**UR**	**6.6×10^{-7}**	**Darby et al. 2005**	0	2A

This table is reproduced with permission from the copyright holder of Hänninen and Knol, 2011.

References to national exposure data sources

For characterizing the population exposures in each country to benzene, formaldehyde, dioxins and lead participating national teams reviewed various national studies.

For the exposure to benzene the outdoor levels from the publicly available air quality database system (AirBase) of the European Environment Agency were considered in conjunction with national studies on indoor sources and levels. Formaldehyde exposures were characterized using data on residential indoor concentrations. The dioxin (including furans and dioxin-like PCBs) exposures were evaluated against WHO mother's milk data, while the final exposures were quantified as daily intakes based on national estimates.

Lead exposures were characterized as blood lead concentrations building on national human biomonitoring projects assuming a log-normal exposure distribution in each country. The references for the national data sources are listed below. Additional information on the application of national exposure data can be found in (Hänninen & Knol, 2011).

Benzene

Belgium

Ballesta PP, Field RA, Connolly R, Cao N, Caracena AB, de Saeger E. 2006. Population exposure to benzene: One day cross-sections in six European cities . Atmospheric Environment 40(18):3355-3366

Cocheo V, Boaretto C, Sacco P, De Saeger E, Ballesta PP, Skov H et al. 2000. Urban benzene and population exposure. Nature 404:141–142.

Finland

Edwards R, Jantunen M. 2001. Benzene exposure in Helsinki, Finland. Atmospheric Environment 35 (2001) 1411-1420.

Hänninen OO, Alm S, Katsouyanni K, Künzli N, Maroni M, Nieuwenhuijsen MJ, Saarela K, Srám RJ, Zmirou D, Jantunen MJ. 2004. The EXPOLIS Study: Implications for exposure research and environmental policy in Europe. Journal of Exposure Analysis and Environmental Epidemiology 14: 440-456.

Jantunen MJ, Hänninen OO, Katsouyanni K, Knöppel H, Künzli N, Lebret E, Maroni M, Saarela K, Srám RJ, Zmirou D. 1998. Air pollution exposure in European cities: The EXPOLIS-study. Journal of Exposure Analysis and Environmental Epidemiology 8 (4): 495-518.

France

OQAI. 2006. Campagne nationale logements : etat de la qualite de l'air dans les logements Français, rapport final, http://www.air-interieur.org/userdata/documents/Document_133.pdf (accessed 19 February 2010)

Germany

Schulz C, Conrad A, Becker K, Kolossa-Gehring M, Seiwert M, Seifert B. 2007. Twenty years of the German Environmental Survey (GerES): Human biomonitoring – Temporal and spatial (West Germany/East Germany) differences in population exposure. International Journal of Hygiene and Environmental Health 210(3-4):271-297.

Italy

ISPRA. 2007. Institute for Environmental Protection and Research. Summary of benzene levels from the National monitoring network.

Carrer P, Maroni M, Alcini D, Cavallo D, Fustinoni S, Lovato L, Visigalli F. 2000. Assessment through environmental and biological measurements of total daily exposure to volatile organic compounds of office workers in Milan, Italy. Indoor Air, 10: 258-268.

Maroni M, Carrer P, Cavallo D, Alcini D, Lovato L, Righetto L. 1998. Daily personal exposure to air pollutants of office workers in Milano. Epidemiology, Vol. 9, N. 4S: 289.

The Netherlands

Van Dongen, Vos. 2007. Gezondheidsaspecten van 1240 woningen, TNO rapport 2007-D-R0188/A

Dioxins (including furans and dioxin-like PCBs)

Belgium

Bilau M, Matthys C, Baeyens W, Bruckers L, De Backer G, Den Hond E, Keune H, Koppen G, Nelen V, Schoeters G, Van Larebeke N, Willems JL, De Henauw S. 2008. Dietary exposure to dioxin-like compounds in three age groups: results from the Flemish environment and health study, Chemosphere, 70, 584-592.

Finland

Kiviranta H. 2005. Exposure and human PCDD/F body burden in Finland [dissertation]. Helsinki, National Public Health Institute.(http://www.ktl.fi/attachments/suomi/julkaisut/julkaisusarja_a/2005/2005a14.pdf, [accessed 23 March 2007].

Kiviranta H, Tuomisto JT, Tuomisto J, Tukiainen E, Vartiainen T. 2005. Polychlorinated dibenzo-p-dioxins, dibenzofurans, and biphenyls in the general population in Finland. Chemosphere, 60(7): 854–869.

France

Fréry N, Volatier J, Zeghnoun A, Falq G, Mouajjah S, Thébault A, Pascal M, Bérat B, Grange D, de Crouy-Chanel P, Sarter H, Heyman C, Guillois-Becel Y, Lucas N, Mathieu A, Noury U, Pouey J, Schmitt M, Salines G. 2006. Dioxins Levels in People Living Around Municipal Solid Waste Incinerators in France. Epidemiology 17(6):S298.

Germany

Umweltbundesamt Webpage„Dioxine. http://www.umweltbundesamt.de/chemikalien/dioxine.htm [accessed: 2014-01-28]

Umweltbundesamt. 2010.Hintergrund – Dioxine“ http://www.dioxindb.de/dokumente/FFE49d01.pdf

Italy

Fattore E, Fanelli R, Turrini A, di Domenico A. 2006. Current dietary exposure to polychlorodibenzo-p-dioxins, polychlorodibenzofurans, and dioxin-like polychlorobiphenyls in Italy. Mol Nutr Food Res. 50(10):915-21. http://www3.interscience.wiley.com/cgi-bin/fulltext/113376466/PDFSTART

The Netherlands

De Mul, A, Bakker MI, Zeilmaker MJ, Traag WA, van Leeuwen SPJ, Hoogenboom RLAP, Boon PE, van Klaveren JD. 2008. Dietary exposure to dioxins and dioxin-like PCBs in The Netherlands anno 2004,. in: Regulatory Toxicology and Pharmacology 51 278–287.

Second hand smoke

International sources

EC. 2009.. Survey on Tobacco. Analytical report. European Commission Flash Eurobarometer 253. 98 pp. http://ec.europa.eu/public_opinion/flash/fl_253_en.pdf (accessed 2010-01-29).

ECRHS. 2002. "The European Community Respiratory Health Survey II." Eur Respir J **20**(5): 1071-9.

Janson C, Kunzli N, et al. 2006. Changes in active and passive smoking in the European Community Respiratory Health Survey. Eur Respir J **27**: 517-524.

Belgium

Swaans M, Spruyt R, Bormans L, Verbeke D, Poelmans E, Goelen F, Geyskens R, Swinnen. 2008. Onderzoek naar degezondheidskwaliteit van Vlaamse woningen. Eindverslag. Studie uitgevoerd in opdracht van Toezicht Volksgezondheid. 2008/MIM/R/137

Finland

Finnish National Public Health Institute. 2004. Health Behaviour and health among the Finnish adult population. Available at: http://www.ktl.fi/attachments/suomi/julkaisut/julkaisusarja_b/2004b13.pdf [Accessed November 4, 2009].

Jousilahti P, Helakorpi S. 2002. Prevalence of exposure to environmental tobacco smoke at work and at home--15-year trends in Finland. Scand J Work Environ Health. 2002;28 Suppl 2:16-20.

Lund KE, Skrondal A, et al. 1998. Children's residential exposure to environmental tobacco smoke varies greatly between Nordic countries. Scand J Soc Med 26: 115-120.

France

INPES. 2005. Institut National de Prévention et d'Education pour la Santé. Baromètre santé 2005. Attitudes et comportements de santé. 2005. Available at: http://www.inpes.sante.fr/Barometres/BS2005/pdf/BS2005_Tabac.pdf [Accessed November 4, 2009].

Germany

Conrad A, Seiwert M, Schulz C, et al. 2008. German Environmental Survey IV: environmental tobacco smoke exposure of German children. International Journal of Environment and Health. 2008;2:397-409.

Schulze A, Lampert T. 2006. Beiträge zur Gesundheitsberichterstattung des Bundes Bundes-Gesundheitssurvey: Soziale Unterschiede im Rauchverhalten und in der Passivrauchbelastung in Deutschland. Robert Koch-Institut; 2006. Available at: http://www.rki.de/cln_178/nn_199884/DE/Content/GBE/Gesundheitsberichterstattung/GBEDownloadsB/Rauchen,templateId=raw,property=publicationFile.pdf/Rauchen.pdf [Accessed November 5, 2009].

Italy

Tominz R, Binkin N, Perra A, Gruppo di lavoro ICONA. 2005. Esposizione al fumo passivo di tabacco nei bambini italiani al secondo anno di vita [Italian chldren's exposure to environmental tobacco smoke in second year of life]. Medico e Bambino. 2005;XXIV(10):683.

The Netherlands

van Gelder BM, Blokstra A, Feenstra TL. 2008. Environmental tobacco smoke in the Netherlands. RIVM; 2008. Available at: http://www.rivm.nl/bibliotheek/rapporten/260601005.pdf [Accessed November 5, 2009]

Formaldehyde

Belgium

Swaans M, Spruyt R, Bormans L, Verbeke D, Poelmans E, Goelen F, Geyskens R, Swinnen. 2008. Onderzoek naar degezondheidskwaliteit van Vlaamse woningen. Eindverslag. Studie uitgevoerd in opdracht van Toezicht Volksgezondheid. 2008/MIM/R/137

Finland

Jurvelin J, Vartiainen M, Jantunen M, Pasanen P. 2001. Personal exposure levels and microenvironmental concentrations of formaldehyde and acetaldehyde in the Helsinki metropolitan area, Finland. Journal of the Air & Waste Management Association, 51(1):17-24.

France

OQAI. 2006. Campagne Nationale Logements : Etat De La Qualite De L'air Dans Les Logements Français, Rapport Final, http://www.air-interieur.org/userdata/documents/Document_133.pdf (accessed 19 February 2010)

Germany

Umweltbundesamt. 2008. Vergleichswerte für flüchtige organische Verbindungen (VOC und Aldehyde) in der Innenraumluft von Haushalten in Deutschland. Bundesgesundheitsbl – Gesundheitsforsch – Gesundheitsschtz 51:109-112.

Italy

Lovreglio P, Carrus A, Iavicoli S, Drago I, Persechino B, Soleo L. 2009. Indoor formaldehyde and acetaldehyde levels in the province of Bari, South Italy, and estimated health risk. J Environ Monit. 11(5):955-61

The Netherlands

Dongen van J, Vos H. 2007 Gezondheidsaspecten van woningen in Nederland, TNO.

Jongeneel R et al. 2009. Binnenmilieu; recente wetenschappelijke ontwikkelingen en beleid op een rij, RIVM

Lead

Belgium

Schroijen C, Baeyens W, Schoeters G, Den Hond E, Koppen G, Bruckers L, Nelen V, Van De Mieroop E, Bilau M, Covaci A, Keune H, Loots I, Kleinjans J, Dhooge W, Van Larebeke N. 2008. Internal exposure to pollutants measured in blood and urine of Flemish adolescents in function of area of residence, Chemosphere, 71, 1317-1325.

Finland

Louekari K. 1991. Estimation of dietary intake of heavy metals-comparison and reliability of methods. University of Kuopio, Alkuperaistutkimukset 1991;4, Kuopio, Finland.

Pönkä. 1998. Lead in the ambient air and blood of children in Helsinki. The Science of the Total Environment 219:1-5.

Soininen L, Mussalo-Rauhamaa H, Hyvönen S. 2005. AMAP Human Health - Biomonitoring and Studying the Nutrition of Mothers and Newborn in the Finnish Lapland. Regional Environmental Publications 389. ISBN 952-11-2000-2, ISBN 952-11-2001-0 (PDF). 110 pp. Rovaniemi, Finland. http://www.environment.fi/default.asp?contentid=162162&lan=en (accessed: 2009-06-10)

TTL. 2006. Biologisen monitoroinnin palveluanalytiikan vuositilasto 2005. Työympäristötutkimuksen raporttisarja 21. http://www.ttl.fi/NR/rdonlyres/4A462F1D-1DD4-45E1-B55F- 19F70FAD89D6/0/bm_vuositilasto05.pdf

France

Falq G, Zeghnoun A, Pascal M, Verhay M, Garnier R, Castetbon K, Olichon D, Féry N. 2008. ENNS : The French Nutrition & Health Survey – Exposure of the French population to lead. Poster at the Fourth International Conference on Metals and Genetics, Descartes University, Paris, July 21-24.

Germany

National human biomonitoring data from the German Environmental Specimen Bank (Umweltprobenbank des Bundes, UPB), available at: http://www.umweltprobenbank.de (accessed: 2009-06-24)

Italy

Apostoli P, Baj A, Bavazzano P, Ganzi A, Neri G, Ronchi A, Soleo L, Di LL, Spinelli P, Valente T, Minoia C. 2002. Blood lead reference values: the results of an Italian polycentric study. Sci Total Environ. 287(1-2):1-11. http://www.sciencedirect.com/science?_ob=MImg&_imagekey=B6V78-442HPW2-1-5&_cdi=5836&_user=2754627&_orig=search&_coverDate=03%2F15%2F2002&_sk=997129998&view=c&wchp=dGLzVlz-zSkzk&md5=4fe31fb05ce747afdbfe3f39019ff751&ie=/sdarticle.pdf

The Netherlands

Peeters E. 2006. Kinderlood; onderzoek naar de loodbloedconcentraties in bloed van Rotterdamse kinderen anno 2005 en de invloed hierop van lood in de bodem, GGD Rotterdam e.o.
